# Supplementary material for: Reaching alignment-profile-based accuracy in predicting protein secondary and tertiary structural properties without alignment
Source: Sci Rep. 2022 May 9;12:7607. doi: 10.1038/s41598-022-11684-w (PMC9085874; doi:10.1038/s41598-022-11684-w)
Supplement: Supplementary file 1 — Supplementary Information. [file 41598_2022_11684_MOESM1_ESM.pdf]

Supplementary Material: Achieving Alignment-profile-based Accuracy in Prediction Protein Secondary and Tertiary Structural Properties without Alignment.

Jaspreet Singh[1,*], Kuldip Paliwal[1,*], Thomas Litfin[1], Jaswinder Singh[1], and Yaoqi Zhou[2,3,4,*]

[1]Signal Processing Laboratory, School of Engineering and Built Environment, Griffith University, Brisbane, QLD 4111, Australia
[2]Institute for Glycomics, Griffith University, Parklands Dr. Southport, QLD 4222, Australia
[3]Institute for Systems and Physical Biology, Shenzhen Bay Laboratory, Shenzhen 518055, China
[4]Peking University Shenzhen Graduate School, Shenzhen 518055, P.R.China
[*]Correspondence to jaspreetsingh2@griffithuni.edu.au, K.paliwal@griffith.edu.au and zhouyq@szbl.ac.cn

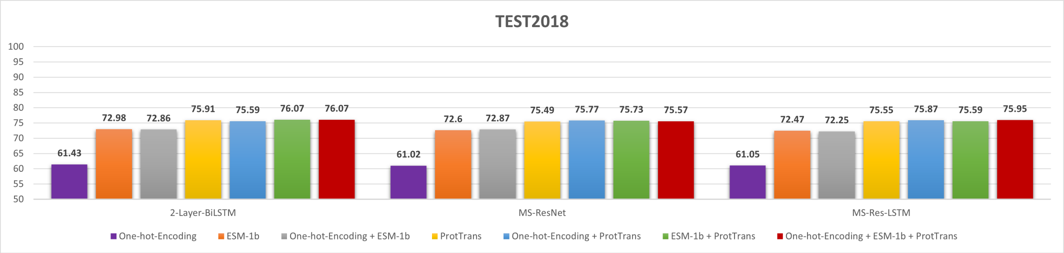

(a)

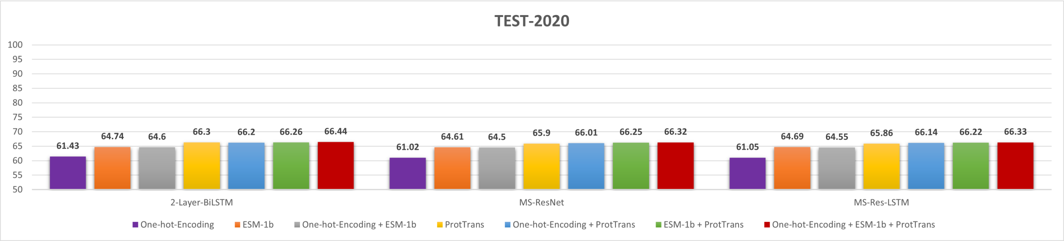

(b)

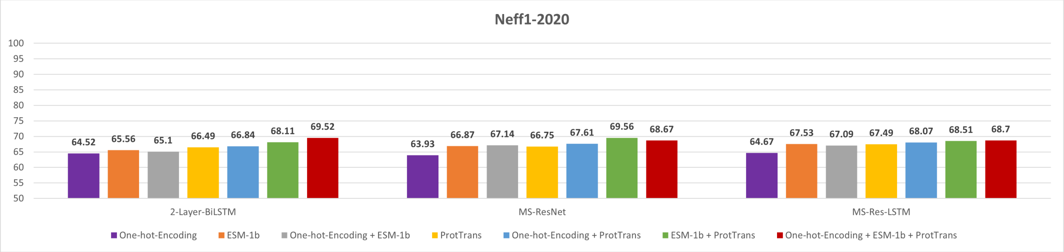

(c)

Supplementary Figure S1: Performance in eight-state (SS8) secondary structure prediction by using different input features as labelled for three different model architectures on three test sets (TEST2018, TEST2020, and Neff1-2020). Performance measure is accuracy for SS8 prediction.

Supplementary Table S1: Performance comparison of different architectures based on different input features for ASA, HSE-u, HSE-d, and CN predictions using TEST2018, TEST2020, and Neff1-2020 sets. Performance measures for ASA, HSE-u, HSE-d, and CN is the Pearson correlation coefficient (PCC).

Features	Model	TEST2018				TEST2020				Neff1-2020			
		ASA	HSE-u	HSE-d	CN	ASA	HSE-u	HSE-d	CN	ASA	HSE-u	HSE-d	CN
One-Hot Encoding	2-Layer-BiLSTM	0.668	0.547	0.549	0.573	0.637	0.387	0.466	0.482	0.653	0.368	0.502	0.519
	MS-ResNet	0.670	0.554	0.551	0.577	0.643	0.390	0.474	0.485	0.656	0.358	0.524	0.545
	MS-Res-LSTM	0.670	0.554	0.551	0.577	0.644	0.390	0.474	0.485	0.657	0.357	0.524	0.545
One-Hot-Encoding + ESM-1b	2-Layer-BiLSTM	0.788	0.717	0.722	0.766	0.702	0.475	0.558	0.578	0.694	0.427	0.588	0.603
	MS-ResNet	0.780	0.710	0.717	0.742	0.703	0.469	0.560	0.587	0.705	0.430	0.584	0.612
	MS-Res-LSTM	0.784	0.717	0.724	0.758	0.705	0.481	0.568	0.590	0. ‘707	0.427	0.586	0.621
One-Hot-Encoding + ProtTrans	2-Layer-BiLSTM	0.808	0.744	0.760	0.785	0.723	0.512	0.592	0.609	0.723	0.469	0.614	0.628
	MS-ResNet	0.803	0.736	0.737	0.764	0.718	0.505	0.585	0.603	0.716	0.461	0.587	0.619
	MS-Res-LSTM	0.803	0.738	0.747	0.783	0.721	0.512	0.591	0.617	0.718	0.457	0.602	0.626
ProtTrans + ESM-1b + One-Hot-Encoding	2-Layer-BiLSTM	0.812	0.745	0.755	0.788	0.730	0.516	0.591	0.612	0.731	0.472	0.613	0.634
	MS-ResNet	0.807	0.748	0.748	0.786	0.722	0.510	0.588	0.607	0.719	0.444	0.599	0.622
	MS-Res-LSTM	0.806	0.749	0.748	0.778	0.723	0.512	0.584	0.606	0.724	0.461	0.597	0.605

Supplementary Table S2: Performance comparison of different architectures based on different input features for ψ, ϕ, θ, and τ predictions on TEST2018, TEST2020, and Neff1-2020 set. Performance measures for ψ, ϕ, θ, and τ is the mean absolute error (MAE).

Features	Model	TEST2018				TEST2020				Neff1-2020			
		ψ	ϕ	θ	τ	ψ	ϕ	θ	τ	ψ	ϕ	θ	τ
One-hot Encoding	2-Layer-BiLSTM	41.603	22.387	9.500	43.258	44.985	23.192	9.971	44.241	43.124	21.220	9.509	41.734
	MS-ResNet	41.307	22.509	9.532	43.382	41.307	44.202	22.766	9.904	41.548	20.717	9.286	40.657
	MS-Res-LSTM	41.345	22.507	9.532	43.480	44.207	22.764	9.904	43.880	41.516	20.715	9.288	40.617
One-Hot-Encoding + ESM-1b	2-Layer-BiLSTM	27.393	17.478	7.160	28.673	39.067	21.350	9.134	38.641	38.162	19.849	8.881	37.955
	MS-ResNet	27.737	17.675	7.247	29.139	38.316	21.281	8.964	37.881	37.832	19.704	8.726	37.338
	MS-Res-LSTM	27.650	17.506	7.200	28.966	38.463	21.310	8.988	38.069	37.803	19.605	8.793	37.580
ProtTrans + One-Hot-Encoding	2-Layer-BiLSTM	24.301	16.245	6.554	25.118	37.600	21.054	8.893	36.819	39.598	20.163	9.129	38.250
	MS-ResNet	24.477	16.396	6.613	25.336	37.281	20.945	8.781	36.443	37.933	19.859	8.753	36.494
	MS-Res-LSTM	24.680	16.368	6.618	25.450	37.452	20.966	8.844	36.620	37.736	19.764	8.798	36.374
ProtTrans + ESM-1b + One-Hot-Encoding	2-Layer-BiLSTM	23.964	16.140	6.540	24.826	36.792	20.668	8.738	36.154	36.846	19.491	8.572	36.032
	MS-ResNet	24.285	16.217	6.577	25.119	37.040	20.877	8.793	36.200	37.211	19.824	8.671	36.324
	MS-Res-LSTM	24.396	16.428	6.617	25.285	36.877	20.846	8.725	36.124	36.746	19.420	8.569	35.488

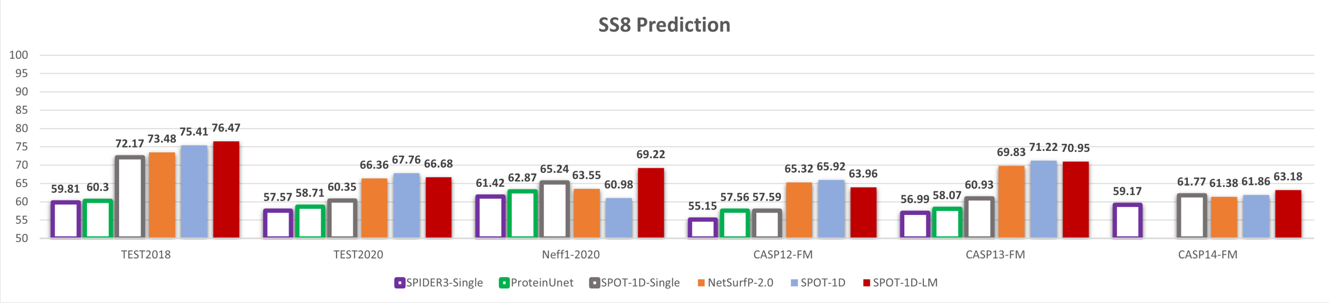

Supplementary Figure S2: Comparing the accuracy of eight-state (SS8) secondary structure prediction of SPOT-1D-LM (this work) with single-sequence-based methods (SPIDER3-Single, ProteinUnet, and SPOT-1D-Single) and sequence-profile-based methods (SPOT-1D and NetSurfP-2.0) on six test sets (TEST2018, TEST2020, NEFF1-2020, CASP12-FM, CASP13-FM, and CASP14-FM).

Supplementary Table S3: P-test comparison of SPOT-1D, NetSurfP-2.0, SPIDER3-Single, ProteinUnet, and PSIPRED-Single w.r.t. SPOT-1D-LM's performance on TEST2018, TEST2020, Neff1-2020, CASP12-FM, CASP13-FM, and CASP14-FM for prediction of secondary structure in three (SS3) and eight (SS8) states.

Models	TEST2018		TEST2020		Neff1-2020		CASP12-FM		CASP13-FM		CASP14-FM	
	SS3	SS8	SS3	SS8	SS3	SS8	SS3	SS8	SS3	SS8	SS3	SS8
SPOT-1D	5.20×10^{-1}	1.50×10^{-1}	-	-	2.77×10^{-5}	2.18×10^{-5}	-	-	-	-	7.4×10^{-1}	4.9×10^{-1}
NetSurfP-2.0	1.40×10^{-3}	2.33×10^{-7}	2.64×10^{-2}	3.40×10^{-1}	1.41×10^{-7}	1.09×10^{-6}	-	-	-	-	3.4×10^{-1}	4.4×10^{-1}
SPOT-1D-Single	3.02×10^{-47}	6.11×10^{-51}	7.08×10^{-5}	1.20×10^{-5}	1.24×10^{-55}	4.22×10^{-53}	1.60×10^{-2}	3.16×10^{-3}	5.29×10^{-6}	2.04×10^{-6}	1.6×10^{-1}	1.6×10^{-1}
SPIDER3-Single	1.38×10^{-54}	4.57×10^{-58}	4.07×10^{-78}	2.85×10^{-79}	1.60×10^{-7}	1.00×10^{-7}	1.10×10^{-3}	2.36×10^{-4}	7.22×10^{-7}	9.05×10^{-6}	4.1×10^{-2}	3.1×10^{-2}
ProteinUnet	1.79×10^{-59}	1.82×10^{-57}	1.47×10^{-73}	8.12×10^{-73}	2.42×10^{-7}	1.02×10^{-6}	9.80×10^{-3}	1.43×10^{-3}	1.23×10^{-6}	1.04×10^{-5}	-	-
PSIPRED-Single	1.64×10^{-68}	-	4.27×10^{-101}	-	1.31×10^{-7}	-	7.10×10^{-4}	-	9.53×10^{-6}	-	6.2×10^{-2}	-

Supplementary Table S4: Comparing the accuracy of SPOT-1D-LM (this work) with single-sequence-based methods (SPIDER3-Single, ProteinUnet, and SPOT-1D-Single) and sequence-profile-based methods (SPOT-1D and NetSurfP-2.0) for prediction of secondary structure in three (SS3) and eight (SS8) states, solvent accessibility (ASA), half-sphere-exposure-up (HSE-u), HSE-down (HSE-d), contact number (CN), backbone angles (ψ, ϕ, θ and τ) for CASP12-FM. Performance measures are accuracy for SS3 and SS8, correlation coefficient for ASA, HSE-u, HSE-d, and CN, and mean absolute errors for the angles.

Model	SS3	SS8	ASA	HSE-u	HSE-d	CN	ψ	ϕ	θ	τ
SPIDER3-Single	69.15	55.15	0.586	0.516	0.467	0.554	47.462	26.168	11.639	47.591
ProteinUnet	71.33	57.56	0.582	0.523	0.482	0.556	46.527	25.942	10.949	46.259
SPOT-1D-Single	72.44	57.59	0.612	0.556	0.522	0.599	43.457	25.426	10.278	44.022
NetSurfP-2.0 (profile)	78.36	65.32	0.668	-	-	-	35.127	22.262	-	-
SPOT-1D (profile)	79.53	65.92	0.667	0.660	0.621	0.692	33.962	21.844	8.700	33.114
SPOT-1D-LM (This work)	77.45	63.96	0.674	0.661	0.629	0.686	35.955	22.290	8.870	35.236

Supplementary Table S5: Comparing the accuracy of SPOT-1D-LM (this work) with single-sequence-based methods (SPIDER3-Single, ProteinUnet, and SPOT-1D-Single) and sequence-profile-based methods (SPOT-1D and NetSurfP-2.0) for prediction of secondary structure in three (SS3) and eight (SS8) states, solvent accessibility (ASA), half-sphere-exposure-up (HSE-u), HSE-down (HSE-d), contact number (CN), backbone angles (ψ, ϕ, θ and τ) for CASP13-FM. Performance measures are accuracy for SS3 and SS8, correlation coefficient for ASA, HSE-u, HSE-d, and CN, and mean absolute errors for the angles.

Model	SS3	SS8	ASA	HSE-u	HSE-d	CN	ψ	ϕ	θ	τ
SPIDER3-Single	71.26	56.99	0.565	0.462	0.408	0.495	46.164	25.315	11.076	46.348
ProteinUnet	70.63	58.07	0.571	0.480	0.433	0.513	46.884	25.036	10.380	46.093
SPOT-1D-Single	73.21	60.93	0.572	0.489	0.464	0.531	45.231	25.124	9.889	44.903
NetSurfP-2.0(profile)	82.11	69.83	0.698	-	-	-	31.817	21.577	-	-
SPOT-1D (profile)	83.55	71.22	0.701	0.683	0.632	0.704	28.489	20.238	7.551	27.867
SPOT-1D-LM (This work)	82.15	70.95	0.763	0.753	0.711	0.785	30.680	20.092	7.563	29.634

Supplementary Table S6: Performance comparison of SPOT-1D-LM with ProtTrans and ESM-1b downstream trained model on CASP12 and NEW364 datasets from ProtTrans. The values provided below are the percentage accuracy of three-state secondary structure secondary structure (SS3) prediction.

Dataset	SPOT-1D-LM	ProtTrans (ProtT5-XL-U50)	ESM-1B
CASP12	82.37	81.4	76.9
NEW364	85.56	84.8	82.6

Supplementary Table S7: Comparing the accuracy of SPOT-1D-LM (this work) with single-sequence-based methods (SPIDER3-Single, ProteinUnet, and SPOT-1D-Single) and sequence-profile-based methods (SPOT-1D and NetSurfP-2.0) secondary structure in three (SS3) and eight (SS8) states, solvent accessibility (ASA), half-sphere-exposure-up (HSE-u), half-sphere-exposure-down (HSE-d), contact number (CN), backbone angles (ψ, ϕ, θ, and τ) for TEST2020-HQ. Performance measures are accuracy for SS3 and SS8, correlation coefficient for ASA, HSE-u, HSE-d, and CN, and mean absolute errors for the angles.

Model	SS3	SS8	ASA	HSE-u	HSE-d	CN	ψ	ϕ	θ	τ
SPIDER3-Single	71.02	58.23	0.612	0.451	0.469	0.504	44.051	24.264	13.061	47.424
ProteinUnet	71.28	58.78	0.573	0.459	0.477	0.499	43.282	23.699	12.120	46.484
SPOT-1D-Single	72.22	59.70	0.627	0.491	0.530	0.540	42.624	23.011	11.466	45.393
NetSurfP-.2.0 (Profile)	80.69	68.34	0.716	-	-	-	31.298	19.904	-	-
SPOT-1D (profile)	81.97	70.41	0.720	0.625	0.679	0.706	28.875	18.782	9.512	32.311
SPOT-1D-LM (This work)	79.70	67.73	0.755	0.624	0.666	0.704	32.011	19.523	9.743	35.271

Supplementary Table S8: Comparing the accuracy of SPOT-1D-LM (this work) with single-sequence-based methods (SPIDER3-Single and SPOT-1D-Single) and sequence-profile-based methods (SPOT-1D and NetSurfP-2.0) for prediction of secondary structure in three (SS3) and eight (SS8) states, solvent accessibility (ASA), half-sphere-exposure-up (HSE-u), HSE-down (HSE-d), contact number (CN), backbone angles (ψ, ϕ, θ and τ) for CASP14-FM. Performance measures are accuracy for SS3 and SS8, correlation coefficient for ASA, HSE-u, HSE-d, and CN, and mean absolute errors for the angles.

Models	SS3	SS8	ASA	HSE-u	HSE-d	CN	ψ	ϕ	θ	τ
SPIDER3-Single	71.88	59.14	0.653	0.560	0.488	0.547	43.844	22.958	10.665	46.976
SPOT-1D-Single	74.70	61.77	0.659	0.584	0.547	0.575	42.184	21.442	8.986	44.643
NetSurfP-2.0 (Profile)	74.30	61.38	0.683	-	-	-	41.181	21.627	-	-
SPOT-1D (Profile)	75.66	61.86	0.669	0.613	0.564	0.595	40.585	21.104	8.799	42.602
SPOT-1D-LM (this work)	76.54	63.18	0.690	0.625	0.582	0.629	40.054	20.861	8.522	42.036

Supplementary Table S9: Inference time comparison for feature generation and neural networks on 250 proteins of TEST2018.

Feature Generation Time Comparison			Neural Network Time comparison		
**Features**	**CPU**	**GPU**	**Model**	**CPU**	**GPU**
PSSM	9.3 hours	-	SPOT-1D	1.1 hours	0.18 hours
HHM	6.9 hours	-	NetSurfP2.0	0.23 hours	-
ProtTrans	0.12 hours	0.01 hours	SPOT-1D-Single	0.04 hours	0.01 hours
ESM-1b	0.08 hours	0.01 hours	SPOT-1D-LM	0.29 hours	0.04 hours
